# Clinical Impact and Tolerance of Unsedated Esophagogastroduodenoscopy in Patients Aged ≥75 Years

**DOI:** 10.1055/a-2901-5915

**Published:** 2026-07-08

**Authors:** Bathilde Leclair, Hélène Levassort, Marion Pepin, Bruno Sawczynski, Laurent Lechowski, Marie-Astrid Desoutter, Thomas Bazin

**Affiliations:** 1 Geriatrics DepartmentClaude Bernard University, Lyon 1, Croix-Rousse HospitalLyonAuvergne-Rhône-AlpesFrance; 2 Geriatric Department, University Medical Department 1AP-HP, Ambroise Paré Hospital, University Hospital Group Paris SaclayBoulogne-BillancourtÎle-de-FranceFrance; 3 Clinical Epidemiology Team, Centre for Research in Epidemiology and Population HealthParis-Saclay University, Inserm U1018, Versailles Saint-Quentin UniversityParisÎle-de-FranceFrance; 4 Medical Information Department (DIM)Paris-Saclay University, AP-HP, Ambroise Paré HospitalParisÎle-de-FranceFrance; 5 Geriatrics DepartmentParis-Saclay University, UVSQ, AP-HP, Ste Périne HospitalBoulogne-BillancourtÎle-de-FranceFrance; 6 Geriatrics DepartmentParis-Saclay University, UVSQ, AP-HP, Ste Périne HospitalParisÎle-de-FranceFrance; 7 Department of Gastroenterology and Nutritional Support, Center for Intestinal Failure, Reference Centre for Rare Diseases MarDI 55100 Beaujon Hospital, AP-HP, Université Paris CitéClichyÎle-de-FranceFrance; 8 Infection and Inflammation Research Unit (UMR 1173), Inserm, UVSQ 27048 Université Paris-SaclayGif-sur-YvetteÎle-de-FranceFrance

**Keywords:** geriatric inpatients, esophagogastroduodenoscopy, unsedated endoscopy, diagnostic yield, treatment impact, procedure tolerance

## Abstract

**Purpose** Esophagogastroduodenoscopy (EGD) is widely used in older adults, but concerns persist about tolerance when performed without anesthesia, especially in very old and frail inpatients. We aimed to assess the diagnostic yield, impact on management, and tolerance of unsedated EGD in hospitalized patients aged ≥75 years.

**Methods** We conducted a retrospective, single-center study including all inpatients aged ≥75 years who underwent EGD without general anesthesia or systemic sedation between January 2018 and December 2019. The primary endpoint was the presence of a macroscopic lesion consistent with the indication. Secondary endpoints were the contribution of EGD to diagnostic and therapeutic management and endoscopist-rated tolerance (good, intermediate, poor).

**Results** Two hundred patients were included (mean age, 86.8 ± 5.8 years; 53% women; 85% malnourished). A macroscopic lesion consistent with the indication was found in 44% of cases. Overall, EGD was contributory in 83.5% of patients, including 70.5% of those without macroscopic lesions, mainly through treatment changes or additional investigations. The completion rate was 83.5%. Tolerance was rated as good in 46.5%, intermediate in 17.5% and poor in 29.5%. In multivariable analysis, a lower Mini-Mental State Examination score and severe malnutrition remained associated with poor tolerance.

**Conclusion** In very old inpatients, unsedated EGD remains highly contributory, even when no macroscopic lesion is observed. Simple clinical factors can help anticipate tolerance and support selective, individualized decision-making regarding EGD in geriatric practice.

## Introduction

Esophagogastroduodenoscopy (EGD) is widely used to investigate upper gastrointestinal symptoms (e.g., dysphagia, epigastric pain, heartburn) and laboratory abnormalities (e.g., iron- or vitamin B12–deficiency anemia), and to perform surveillance for selected conditions (e.g., cirrhosis, celiac disease). Each year, an estimated 6.1 million upper endoscopies are performed in the United States. 1 When performed without general anesthesia (GA), EGD remains a brief, fasting-only procedure with fewer anesthesia-related risks than EGD performed under GA. 2

Population aging is increasing demand for upper GI investigations: life expectancy and healthy life expectancy continue to rise worldwide, and the burden of upper GI disease grows with age. 2 3 Prior studies of patients aged ≥75 years suggest acceptable safety and tolerability. 4 5 However, data are limited on two issues of high clinical relevance in very old inpatients: (i) the diagnostic yield aligned with the indication and (ii) the downstream management impact when no macroscopic lesion is identified. In addition, predictors of procedural tolerance in this age group are not well defined.

We therefore evaluated, in a single-center cohort of adults aged ≥75 years undergoing EGD without GA or systemic sedation, the (a) rate of macroscopic findings consistent with the indication, (b) contribution of EGD to diagnostic and therapeutic decision-making, and (c) factors associated with endoscopist-rated tolerance.

## Patients/Material and Methods

### Study Design and Population

We conducted a retrospective, single-center study including all consecutive inpatients aged ≥75 years who underwent EGD at Ambroise Paré Hospital between January 1, 2018 and December 31, 2019. This pre-COVID-19 study period was retained because it reflected routine unsedated EGD practice before pandemic-related changes in endoscopy organization and patient selection. The delay between data collection and publication was related to the initial academic nature of the project and the subsequent methodological and statistical revisions required to prepare the present manuscript. All procedures were performed without general anesthesia or systemic sedation; only topical pharyngeal anesthesia with lidocaine spray was used. At our institution, conscious sedation with benzodiazepines and/or opioids was not available for routine EGD during the study period, which explains the absence of sedated cases in this cohort.

Indications for EGD were jointly validated by the attending physician and the endoscopist. In routine practice, the decision to perform EGD was based on clinical judgment, taking into account the expected diagnostic usefulness of the procedure and the anticipated ability of the patient to tolerate an unsedated examination. No formal standardized selection score was used. This should be considered when interpreting the generalizability of the results. For analysis, indications were categorized into four groups:

1. anemia with overt bleeding (e.g., melena, hematemesis),
2. anemia without overt bleeding,
3. pain, dysphagia, weight loss, or asthenia,
4. cirrhosis assessment or follow-up.

The cohort was characterized by advanced age, substantial comorbidity burden, functional dependency, and a high prevalence of malnutrition, as detailed in Table 1 .

**Table 1:** Patients’ baseline characteristics according to a macroscopic diagnosis consistent with the indication ( *n* = 200).

	NA	Overall population *n* = 200	No macroscopic diagnosis consistent with the indication *n* = 112 (56%)	Macroscopic diagnosis consistent with the indication *n* = 88 (44%)	p*
***Sociodemographic characteristics***					
Age, mean (sd)	0	86.86 (5.82)	86.83 (5.80)	86.89 (5.87)	0.9
Sex, *n* (%)	0				
Women		107 (53.5)	63 (56.2)	44 (50.0)	0.5
Men		93 (46.5)	49 (43.8)	44 (50.0)	
ADL (/6), mean (sd)	0	5.08 (1.29)	5.09 (1.27)	5.07 (1.32)	0.9
IADL (/8), mean (sd)	0	4.01 (2.23)	4.05 (2.23)	3.95 (2.23)	0.8
Place of residence, *n* (%)	0				
Home		182 (91.0)	105 (93.8)	77 (87.5)	0.3
Nursing home		11 (5.5)	4 (3.6)	7 (8.0)	
Senior residence		6 (3.0)	3 (2.7)	3 (3.4)	
Hotel		1 (0.5)	0 (0)	1 (1.1)	
MMSE score, mean (sd)	58	20.77 (6.01)	21.19 (6.09)	20.24 (5.92)	0.4
MMSE > 24		41 (20.5)	25 (22.3)	16 (18.2)	0.7
MMSE ≥18 and ≤24		57 (28.5)	33 (29.5)	24 (27.3)	0.8
MMSE < 18		43 (21.5)	20 (17.9)	23 (26.1)	0.3
***Comorbidities***					
Charlson score (/29), mean (sd)	0	6.86 (1.89)	6.77 (1.69)	6.99 (2.11)	0.4
Malnutrition ^a^ , *n* (%)	0				
No Malnutrition		31 (15.5)	22 (19.6)	9 (10.2)	0.08
Moderate malnutrition		92 (46.0)	53 (47.3)	39 (44.3)	0.8
Severe malnutrition		77 (38.5)	37 (33.0)	40 (45.5)	0.1
***Medications***					
Polypharmacy ^b^ (≥5/d), *n* (%)	0	143 (71.5)	80 (71.4)	63 (71.6)	1.0
Anticoagulants, *n* (%)	0	75 (37.5)	46 (41.1)	29 (33.0)	0.3
Antiplatelets agent, *n* (%)	0	63 (31.5)	36 (32.1)	27 (30.7)	0.9
Proton pump inhibitor, *n* (%)	0	89 (44.5)	56 (50.0)	12 (13.6)	**<0.001**
Psychoactive drug ^bc^ , *n* (%)	0	89 (44.5)	50 (44.6)	39 (44.3)	1.0
Drugs that may delay gastric emptying ^cd^ , *n* (%)	1	72 (36.0)	41 (36.6)	31 (35.2)	0.7
Alcohol use disorder ^e^ , *n* (%)	2	27 (13.5)	13 (11.6)	14 (15.9)	0.7
Smoking ^f^ , *n* (%)	0	45 (22.5)	30 (26.8)	15 (17.0)	0.1
***Biology data***					
Hemoglobin (g/dl), mean (sd)	0	10.2 (1.6)	10.3 (1.7)	10.1 (1.6)	0.4
Mean corpuscular volume (fl), mean (sd)	0	88.0 (8.3)	87.5 (7.8)	88.7 (8.8)	0.3
C-reactive protein (mg/L), mean (sd)	4	42.0 (50.2)	42.9 (48.7)	41.0 (52.3)	0.8
Ferritin (μg/L), mean (sd)	30	284.8 (390.9)	291.9 (429.4)	275.7 (338.6)	0.8

*Student’s *t* -test for continuous variables, χ ^2^ test for categorical variables.

^a^ Malnutrition:

Moderate malnutrition: serum albumin under 35 g/L or BMI under 21 kg/m ^2^ .

Severe malnutrition: serum albumin under 30 g/L or BMI under 18 kg/m ^2^ .

^b^ Polypharmacy: taking more than four medications per day.

^c^ Psychoactive drugs: antidepressants, anxiolytics, thymoregulators, antiepileptics, antipsychotics, and anti-dopaminergics.

^d^ Gastroparesis drug: anticholinergic, opioid, L-DOPA, antidepressant.

^e^ Alcohol use disorder: history of chronic alcohol use disorder, whether or not weaned.

^f^ Smoking: more than 20 pack-years, whether or not weaned.

*Abbreviations:* ADL (Activity of daily living); IADL (Instrumental activities of daily living); MMSE (Mini mental State Examination); NA: not available.

### Data Collection

Eligible patients were identified through the hospital information system (Programme de Médicalisation des Systèmes d’Information, PMSI). Clinical and demographic data were extracted from electronic medical records. The following variables were collected:

- Sociodemographic and functional data: age, sex, place of residence (home, nursing home, senior residence, or other), autonomy assessed using the Activities of Daily Living (ADL, 0–6) and Instrumental ADL (IADL, 0–8) scales. 6 7
- Cognitive status was assessed using the French GRECO version of the Mini-Mental State Examination (MMSE), validated and standardized in France. 8 9 Scores were extracted retrospectively from clinical records, where the MMSE is routinely administered as part of standard care. The MMSE was not specifically administered for research purposes. For a recent synthesis of its diagnostic accuracy, see Arevalo-Rodriguez et al. 10
- Comorbidities: quantified by the Charlson Comorbidity Index (19 items, score range 0–29). 11
- Nutritional status: defined according to the French Haute Autorité de Santé recommendations valid during the study period. Moderate malnutrition was defined as serum albumin <35 g/L or body mass index (BMI) <21 kg/m ^2^ . Severe malnutrition was defined as serum albumin <30 g/L or BMI <18 kg/m ^2^ . 6
- Medication exposure: use of antiplatelet agents, anticoagulants, psychoactive drugs (antidepressants [ATC N06], anxiolytics [ATC N05B], mood stabilizers [ATC N03A], antiepileptics [ATC N03A], antipsychotics [ATC N05A], anti-dopaminergics [ATC N04B]), and drugs that may delay gastric emptying (anticholinergics [ATC R03AL-G04BD], opioids [ATC N02A], L-DOPA [ATC N04B], some antidepressants [ATC N06]). Polypharmacy was defined as taking more than four medications per day. 12 13
- Lifestyle factors: history of alcohol use disorder (whether or not weaned) and smoking >20 pack-years (whether or not weaned).
- Laboratory data: hemoglobin, mean corpuscular volume, C-reactive protein, ferritin, and serum albumin.

### Endpoints

- The primary endpoint was the presence of a macroscopic lesion consistent with the indication for EGD (e.g., bleeding lesion in case of anemia).
- Secondary endpoints were:
  (i) contribution of EGD to patient management, defined as therapeutic modification (initiation or discontinuation of treatment, endoscopic intervention) or proposal for additional diagnostic procedures, and
  (ii) tolerance of EGD, as assessed by the endoscopist.

### Tolerance Assessment

Tolerance was assessed retrospectively from the endoscopy report, based on the routine rating documented by the endoscopist. Tolerance was categorized as good, intermediate, or poor. Good tolerance corresponded to a procedure completed without relevant discomfort recorded in the report. Intermediate tolerance corresponded to a completed procedure with reported discomfort or procedural difficulty, without interruption. Poor tolerance corresponded to marked discomfort and/or interruption of the procedure because of intolerance. The procedure was considered completed when the endoscope reached the second part of the duodenum.

### Statistical Analysis

Patients were categorized according to whether a macroscopic lesion consistent with the indication was observed. Continuous variables were summarized as means (±SD), categorical variables as frequencies (%). Between-group comparisons used Student’s *t* -test for continuous variables and χ ^2^ or Fisher’s exact tests for categorical variables.

Univariate logistic regression analyses were performed to identify associations between baseline characteristics and:

1. diagnostic yield,
2. management contribution,
3. poor tolerance.

To address the potential impact of missing cognitive data, we compared patients with and without available MMSE data. We also compared baseline and procedural characteristics between incomplete and completed EGD procedures. Additionally, we performed a restricted multivariable logistic regression model for poor tolerance. Variables were selected a priori based on clinical relevance and univariate findings and included age per 5-year increase, sex, MMSE score, severe malnutrition, and ADL score. Because MMSE was included in the model, the analysis was performed as a complete-case analysis among patients with available MMSE data. For variables with low event counts, estimates were interpreted cautiously because of possible instability related to sparse data. The alpha risk was set at 0.05. All analyses were performed with R v4.0 (R Foundation for Statistical Computing, Vienna, Austria). 14

### Regulatory and Ethics Compliance

This noninterventional, retrospective study reused deidentified clinical data under the French MR-004 framework (CNIL Deliberation No. 2018-155); patients were informed with an opt-out option; no IRB approval or individual written consent was required.

## Results

### Study Population

Between January 1, 2018 and December 31, 2019, 200 patients aged ≥75 years underwent EGD and were included. The mean age was 86.8 ± 5.8 years (range 75–98), with a slight female predominance (53%). The mean Charlson Comorbidity Index was 6.9 ± 1.9. Functional status was impaired, with mean ADL 5.1/6 ± 1.3 and IADL 4.0/8 ± 2.2. Cognitive status was available for 142 patients: mean MMSE 20.8/30 ± 6.0. Nearly 85% of patients were malnourished: 46% moderately and 38.5% severely ( Table 1 ). MMSE data were available for 142 patients and missing for 58. To assess potential bias related to missing cognitive assessment, we compared baseline characteristics according to MMSE availability. Patients without MMSE assessment were younger than those with available MMSE data (84.69 vs. 87.74 years, *p* < 0.001) and had higher IADL scores (4.91 vs. 3.64, *p* < 0.001). Malnutrition, polypharmacy, the Charlson Comorbidity Index, ADL score, hemoglobin, and C-reactive protein levels did not differ significantly between the groups. Psychoactive drug exposure and drugs that may delay gastric emptying were less frequent in patients without MMSE assessment. These data are presented in **Supplementary Table 1** . MMSE-based analyses were therefore interpreted as exploratory because cognitive data were not available for all patients.

### Indications for EGD

The main indication was anemia, representing approximately 80% of procedures, including 45% with overt bleeding. Sixteen percent of procedures were performed for pain, dysphagia, or impaired general condition, and 4% for cirrhosis work-up. With the exception of cirrhosis assessments, most macroscopic findings consisted of ulcerative or inflammatory lesions such as ulcers or esophagitis ( Table 2 ).

**Table 2:** Macroscopic diagnosis by EGD, consistent with the indication for the procedure.

*Indicationn* (%)	Anemia ^α^ with overt bleeding 31	Anemia ^α^ without overt bleeding 36	Cirrhosis: assessment or follow-up 7	Pain, dysphagia, impaired general condition 14
**Macroscopic diagnosis**				
*Helicobacter pylori* gastritis/Esophageal candidiasis	1 (3.2)	1 (2.8)	0 (0)	3 (21.4)
Ulcer/gastritis/esophagitis Without germs identified	27 (87.1)	31 (86.1)	0 (0)	9 (64.3)
Angiodysplasia/Esophageal varices	3 (9.7)	1 (2.8)	7 (100)	0 (0)
Neoplastic disease	0 (0)	3 (8.3)	0 (0)	2 (14.3)

α: Hemoglobin <12.5 g/dL.

### Diagnostic Yield

A macroscopic lesion consistent with the indication was identified in 88 patients (44%). Two baseline characteristics were associated in univariate analysis with a higher probability of such a diagnosis: absence of proton pump inhibitor (PPI) use ( *p* < 0.001) and indication of cirrhosis work-up ( *p* = 0.03) ( Tables 1 , 3 , and 4 ).

**Table 3:** Contribution of EGD in the diagnosis and therapeutic work-up.

*n* (%)	Overall population *n* = 200	No macroscopic diagnosis consistent with the indication *n* = 112 (56%)	Macroscopic diagnosis consistent with the indication *n* = 88 (44%)	p*
***Indication***				
Anemia ^α^ with overt bleeding	73 (36.5)	42 (37.5)	31 (35.2)	0.09
Anemia ^α^ without overt bleeding	87 (43.5)	51 (45.5)	36 (40.9)	0.6
Pain, dysphagia, impaired general condition	32 (16.0)	18 (16.1)	14 (15.9)	1.0
Cirrhosis: assessment or follow-up	8 (4.0)	1 (0.9)	7 (8.0)	**0.03**
***Referral hospitalization service***				
Gastroenterology department	12 (6.0)	7 (6.2)	5 (5.7)	0.7
Internal medicine department	46 (23.0)	28 (25.0)	18 (20.5)	
Geriatric department	142 (71.0)	77 (68.8)	65 (73.9)	
***Impact on the diagnosis and therapeutic work-up***				
**Contributory EGD** ^ϒ^	167 (83.5)	79 (70.5)	88 (100.0)	**<0.001**
***Diagnosis work-up***				
Proposal for new test	84 (42.0)	75 (67.0)	9 (10.2)	**<0.001**
Endoscopic procedure ^#^	52 (26.0)	47 (42.0)	5 (5.7)	**<0.001**
Sectional imaging ^£^	32 (16.0)	28 (25.0)	4 (4.5)	**<0.001**
***Therapeutic work-up***				
Therapeutic modification	91 (45.5)	11 (9.8)	80 (90.9)	**<0.001**
*Endoscopic treatment*	9 (4.5)	0 (0.0)	9 (10.2)	**0.002**
*Treatment introduction*				
Anticoagulant restart	1 (0.5)	0 (0.0)	1 (1.1)	0.9
Proton pump inhibitor	71 (35.5)	1 (0.9)	70 (79.5)	**<0.001**
Beta blocker	2 (1.0)	0 (0.0)	2 (2.3)	0.4
Fluconazole	4 (2.0)	0 (0.0)	4 (4.5)	0.08
Prokinetic drug	1 (0.5)	0 (0.0)	1 (1.1)	0.9
* Helicobacter pylori* treatment	5 (2.5)	0 (0.0)	5 (5.7)	**0.04**
*Stop Treatment*				
Proton pump inhibitor	11 (5.5)	11 (9.8)	0 (0.0)	**0.007**
***Tolerance***				
Completed EGD	167 (83.5)	85 (75.9)	82 (93.2)	**0.002**
Tolerance				
Good	93 (46.5)	46 (41.1)	47 (53.4)	0.1
Intermediate	35 (17.5)	21 (18.8)	14 (15.9)	0.7
Poor	59 (29.5)	37 (33.0)	22 (25.0)	0.3

α: Hemoglobin <12.5g/dL.

#: Endoscopic procedure: esophagogastroduodenoscopy/colonoscopy/rectosigmoidoscopy/videocapsule/oeso-gastro-duodenal transit.

£: Sectional imaging: CT scan, CT colonography, MRI.

ϒ: Proposed further investigation OR therapeutic change.

*Student’s *t* -test for continuous variables, χ ^2^ test for categorical variables.

*Abbreviations:* EGD (esophagogastroduodenoscopy).

**Table 4:** Clinical features associated with indication-consistent macroscopic diagnosis, management contribution and poor tolerance in unadjusted logistic regression.

	Macroscopic diagnosis consistent with the indication Crude OR [95% CI]	*p*	Contributory EGD ^ϒ^ Crude OR [95% CI]	*p*	Poor tolerance Crude OR [95% CI]	*p*
***Indications***						
Anemia ^α^ with overt bleeding	0.91 [0.50, 1.62]	0.7	1.18 [0.55, 2.68]	0.7	1.58 [0.84, 2.94]	0.2
Anemia ^α^ without overt bleeding	0.83 [0.47, 1.45]	0.9	1.05 [0.50, 2.28]	0.9	0.57 [0.30, 1.06]	0.08
Pain, dysphagia, impaired general condition	0.99 [0.45, 2.11]	0.9	0.52 [0.22, 1.36]	0.2	1.31 [0.57, 2.88]	0.5
Cirrhosis: assessment or follow-up	9.59 [1.66, 181.13]	**0.04**	0 [0, NA]	0.9	0.80 [0.11, 3.54]	0.8
***Sociodemographic characteristics***						
Age (/5 year)	1.01 [0.79, 1.28]	0.9	1.17 [0.85, 1.62]	0.3	1.35 [1.03, 1.81]	**0.03**
Male	1.29 [0.73, 2.26]	0.4	0.91 [0.43, 1.93]	0.8	0.53 [0.28, 0.98]	**0.046**
ADL (/6), per 1 point	0.99 [0.79, 1.23]	0.9	0.86 [0.60, 1.16]	0.3	0.77 [0.61, 0.97]	**0.03**
IADL (/8), per 1 point	0.98 [0.86, 1.11]	0.8	0.86 [0.72, 1.02]	0.09	0.82 [0.71, 0.94]	**0.007**
MMSE score (/30), per 1 point	0.97 [0.92, 1.03]	0.3	0.99 [0.92, 1.07]	0.8	0.93 [0.87, 0.99]	**0.02**
***Comorbidities***						
Charlson score (/29)	1.06 [0.92, 1.24]	0.4	1.01 [0.83, 1.23]	0.9	1.01 [0.86, 1.19]	0.9
Hypothyroïdia	1.11 [0.48, 2.53]	0.8	1.29 [0.42, 3.99]	0.7	1.31 [0.53, 3.08]	0.5
Malnutrition ^a^						
No Malnutrition						
Moderate malnutrition	1.80 [0.77, 4.51]	0.2	1.34 [0.38, 3.35]	0.6	3.68 [1.17, 16.30]	**0.045**
Severe malnutrition	2.64 [1.11, 6.73]	**0.03**	1.19 [0.57, 2.51]	0.6	5.96 [1.89, 26.45]	**0.006**
***Medications***						
Polypharmacy (≥5/d) ^b^	1.01 [0.54, 1.88]	0.9	1.11 [0.47, 2.46]	0.8	0.98 [0.50, 1.95]	0.9
Anticoagulants	0.71 [0.39, 1.26]	0.2	1.06 [0.49, 2.36]	0.9	0.65 [0.33, 1.22]	0.2
Antiplatelets agent	0.93 [0.51, 1.70]	0.8	1.07 [0.49, 2.50]	0.9	0.84 [0.42, 1.61]	0.6
Psychoactive drugs ^c^	0.99 [0.56, 1.73]	0.9	0.83 [0.39, 1.76]	0.6	1.75 [0.95, 3.24]	0.07
Gastroparesis drug ^d^	0.93 [0.52, 1.66]	0.8	0.54 [0.25, 1.16]	0.1	1.37 [0.73, 2.56]	0.3
Alcohol use disorder ^e^	1.45 [0.64, 3.30]	0.4	1.70 [0.55, 7.48]	0.4	0.49 [0.16, 1.28]	0.2
Smoking ^f^	0.56 [0.27, 1.11]	0.1	0.61 [0.27, 1.45]	0.2	0.84 [0.38, 1.72]	0.6
***Biology data***						
Hemoglobin (g/dL)	0.93 [0.78, 1.11]	0.4	0.89 [0.71, 1.11]	0.3	1.17 [0.97, 1.41]	0.09
Mean corpuscular volume (fl)	1.02 [0.98, 1.05]	0.3	0.99 [0.95, 1.04]	0.9	1.02 [0.98, 1.06]	0.3
C-reactive protein (mg/L)	1.00 [0.99, 1.00]	0.8	0.99 [0.99, 1.00]	0.2	1.01 [1.00, 1.01]	**0.005**
Ferritin (μg/L)	1.00 [1.00, 1.00]	0.8	0.99 [0.99, 1.00]	0.2	1.00 [1.00, 1.00]	**0.04**

α: Hemoglobin <12.5 g/dL.

ϒ: Proposed further investigation OR therapeutic change.

^a^ Malnutrition:

Moderate malnutrition: serum albumin under 35 g/L or BMI under 21 kg/m ^2^ .

Severe malnutrition: serum albumin under 30 g/L or BMI under 18 kg/m ^2^ .

^b^ Polypharmacy: taking more than 4 medications per day.

^c^ Psychoactive drugs: antidepressants, anxiolytics, thymoregulators, antiepileptics, antipsychotics, anti-dopaminergics.

^d^ Gastroparesis drug: anticholinergic, opioid, L-DOPA, antidepressant.

^e^ Alcohol use disorder: history of chronic alcohol use disorder, whether or not weaned.

^f^ Smoking: more than 20 pack-years, whether or not weaned.

*Abbreviations* : EGD (esophagogastroduodenoscopy); ADL (Activity of daily living); IADL (Instrumental activities of daily living); MMSE (Mini mental State Examination).

### Impact on Patient Management

**Fig. 1:**
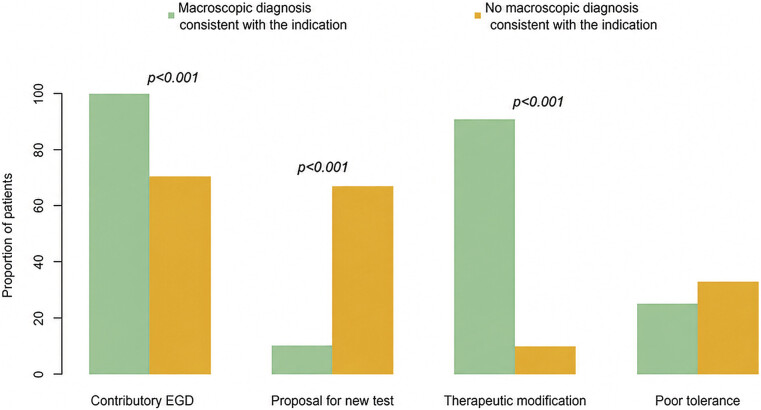
Contribution and tolerance of EGD according to macroscopic diagnosis.

When a consistent macroscopic lesion was identified ( *n* = 88), EGD was contributory in 100% of cases ( Fig. 1 ). A new treatment was introduced in 83 patients (94%), mainly PPI (79.5%), but also *Helicobacter pylori* eradication (5.7%) or anticoagulation (1.1%). A further diagnostic work-up was proposed in nine patients (10.5%) with an indication-consistent macroscopic lesion, including staging or extension assessment of neoplastic disease ( *n* = 4) and repeat EGD under general anesthesia ( *n* = 5). Repeat EGD under general anesthesia was proposed when the index unsedated procedure or the clinical context was considered insufficient for definitive management, particularly in cases of incomplete visualization, poor tolerance, or persistent clinical suspicion requiring a more controlled examination.

Among the 112 patients without an indication-consistent macroscopic diagnosis, EGD was still contributory in 70.5% of cases. It led to discontinuation of unnecessary treatment in 11 patients (9.8%) and to additional investigations in 75 patients (67%). These additional investigations consisted of further endoscopic procedures in 47 patients (42.0%) and sectional imaging in 28 patients (25.0%). The “additional endoscopic procedure” variable was recorded as a composite category in the retrospective database and included repeat upper endoscopy, colonoscopy, rectosigmoidoscopy, videocapsule endoscopy, or upper gastrointestinal contrast study; individual subtypes were not consistently available in the source dataset.

### Tolerance and Completion

The overall completion rate was 83.5% ( Table 5 ). Completion was more frequent among procedures in which a macroscopic diagnosis consistent with the indication was identified (93.2% vs. 75.9%, *p* = 0.002). This association should be interpreted cautiously, as incomplete procedures may have reduced the probability of detecting a relevant macroscopic lesion. Endoscopist-rated tolerance did not differ significantly between groups: good in 46.5%, intermediate in 17.5%, and poor in 29.5% ( Table 3 ). Among the 33 incomplete procedures, the endoscopy reports did not include a standardized field specifying the exact reason for incompletion. Using endoscopist-rated tolerance as the only systematically available procedural information, 21 incomplete EGDs (63.6%) were rated as poorly tolerated, five (15.2%) as intermediately tolerated, 2 (6.1%) as well tolerated, and five (15.2%) had missing or nonclassified tolerance data. Baseline characteristics were broadly similar between patients with incomplete and completed procedures, including age, functional autonomy, MMSE availability and score, malnutrition, polypharmacy, and comorbidity burden. Anticoagulant use was less frequent among patients with incomplete procedures (21.2% vs. 40.7%, *p* = 0.034). These data are presented in **Supplementary Table 2** .

**Table 5:** Contribution of EGD according to indication and tolerance.

*n* (%)	Overall population *n* = 200	Noncontributory EGD *n* = 33 (16.5%)	Contributory EGD *n* = 167 (83.5%)	p*
***Indication***				
Anemia ^α^ with overt bleeding	73 (36.5)	11 (33.3)	62 (37.1)	0.8
Anemia ^α^ without overt bleeding	87 (43.5)	14 (42.4)	73 (43.7)	1.0
Pain, dysphagia, impaired general condition	32 (16.0)	8 (24.2)	24 (14.4)	0.2
Cirrhosis: assessment or follow-up	8 (4.0)	0 (0)	8 (4.8)	**NA**
***Tolerance***				
Completed EGD	167 (83.5)	27 (81.8)	140 (83.8)	**0.9**
Tolerance				
Good	93 (46.5)	17 (42.4)	79 (47.3)	0.7
Intermediate	35 (17.5)	9 (27.3)	26 (15.6)	0.2
Poor	59 (29.5)	8 (24.2)	51 (30.5)	0.6

*Abbreviation* : EGD (esophagogastroduodenoscopy).

α: Hemoglobin <12.5 g/dL

### Predictors of Tolerance

Univariate analyses identified several predictors of tolerance ( Table 4 ).

- Protective factors: male sex (OR = 0.53; *p* = 0.046), higher MMSE score (OR = 0.93 per 1-point increase; *p* = 0.02), greater autonomy on ADL ( *p* = 0.03), and IADL scales ( *p* = 0.007).
- Risk factors: older age (OR = 1.53 per 5-year increase; *p* = 0.03), malnutrition, particularly severe malnutrition (OR = 3.68 for moderate, *p* = 0.045; OR = 5.96 for severe, *p* = 0.006).

In the restricted multivariable model including age, sex, MMSE score, severe malnutrition, and ADL score, MMSE score remained inversely associated with poor tolerance (adjusted OR 0.93 per 1-point increase, 95% CI 0.87-1.00; *p* = 0.048), while severe malnutrition remained associated with higher odds of poor tolerance (adjusted OR 2.49, 95% CI 1.13-5.52; *p* = 0.024). Age, male sex, and ADL score were not independently associated with poor tolerance in this complete-case model. These results are presented in **Supplementary Table 3** .

## Discussion

In this cohort of hospitalized patients aged ≥75 years, EGD identified a macroscopic lesion consistent with the indication in 44% of cases, more frequently in the absence of PPI use or in the setting of cirrhosis. Overall, the procedure was contributory in 83.5% of patients, either by enabling therapeutic modification or prompting further diagnostic work-up.

These findings are in line with previous reports. An Italian series of >3000 patients over 65 years reported macroscopic findings in 49.5% of cases, 15 while a recent single-center study in patients aged >80 years reported only 18%. 4 Compared with these studies, our population was older and frailer, which may explain differences in diagnostic yield. Importantly, unlike prior reports, we only considered lesions consistent with the indication, thereby minimizing incidental findings. This methodological choice underscores the clinical relevance of our results.

The inverse association between PPI use and diagnostic yield was expected, as acid suppression may mask lesions. However, this should not by itself contraindicate EGD when clinically indicated. Furthermore, in our center, indications were validated by both the attending physician and the endoscopist, which likely contributed to the high proportion of contributory procedures. Appropriateness of indications has previously been shown to increase the diagnostic relevance of EGD. 16

Notably, the observed association between completed procedures and a higher diagnostic yield should not be interpreted causally. Rather, incomplete examinations may have decreased the likelihood of identifying a lesion consistent with the indication.

Interestingly, all procedures yielding a macroscopic diagnosis were contributory to subsequent management. Even when no macroscopic lesion was found, nearly 70% of EGDs still influenced patient care, either through treatment adjustment or by directing further investigations. In nearly 10% of these cases, PPI therapy was discontinued, an important benefit given the potential adverse effects of long-term PPI exposure, especially in frail older adults. 17 Thus, EGD not only establishes diagnoses but also rules out suspected pathology and avoids unnecessary treatment, a point rarely highlighted in previous studies.

Regarding tolerance, our study reported no procedure-related adverse events. The overall completion rate was 83.5%, lower than that described in younger populations, but reflecting the advanced age (mean age, 87 years) and high prevalence of frailty and malnutrition in our cohort. Completion was significantly higher when a macroscopic lesion was identified, likely reflecting better cooperation in patients with less discomfort. Previous studies have also reported acceptable safety profiles in older patients, with complication rates ranging from 2 to 5.4 per 1000 procedures. 15 18 Our results align with those of Clarke et al. in patients aged ≥85 years 5 and with a more recent study in patients aged >75 years, in which only 5% experienced nonserious adverse events with light sedation. 4 Procedural incompletion was strongly associated with endoscopist-rated poor tolerance; however, the two outcomes should not be considered interchangeable. Some incomplete procedures were not rated as poorly tolerated, whereas some completed procedures were still rated as poorly tolerated. In addition, the retrospective endoscopy reports did not include a standardized field specifying the reason for incompletion, and interruptions, when documented, could reflect technical or clinical factors in addition to patient intolerance. Completion rate should therefore be interpreted as a procedural outcome distinct from tolerance, particularly in very old patients.

We were also able to identify clinical predictors of tolerance. Poor tolerance was associated with advanced age, dependency, cognitive impairment, and malnutrition—particularly severe malnutrition, which increased the risk nearly sixfold. Conversely, male sex, better cognitive performance, and preserved autonomy were protective. These findings suggest that tolerance can be anticipated, enabling better patient selection and targeted discussion with patients, families, and care teams. In a restricted multivariable model, lower MMSE score and severe malnutrition remained associated with poor tolerance, whereas age, sex, and ADL score were no longer independently associated. These findings support the clinical relevance of cognitive and nutritional assessment before unsedated EGD in older inpatients, while remaining exploratory because the model was based on complete cases with available MMSE data.

Our study has several limitations. Its retrospective design implies missing data, notably on cognitive and nutritional parameters. Newer definitions of malnutrition incorporating sarcopenia and recent weight loss could not be applied. The relatively small sample size limited our ability to detect rare complications. In addition, the contribution of EGD to management may have been influenced by contextual clinical decisions. Tolerance was assessed retrospectively from endoscopist reports and not from a standardized prospective research form. Moreover, patient-reported procedural tolerance was not available. Although endoscopist-rated tolerance is clinically informative, it may not fully reflect the patient’s subjective experience. MMSE data were not available for all patients, which may have introduced selection bias in the analysis of cognitive status as a predictor of tolerance. Missing MMSE values likely reflect the constraints of routine geriatric care rather than a predefined research protocol. An important limitation of our study is selection bias. Because this was a retrospective study of routine clinical practice, patients were referred for EGD based on the judgment of the treating physician and the endoscopist. Patients considered too frail or unlikely to tolerate an unsedated procedure may therefore have been less frequently referred, which may limit generalizability and may have led to an underestimation of poor tolerance. However, this bias reflects real-world interdisciplinary decision-making and highlights the importance of collaboration between geriatricians and gastroenterologists. Finally, the type of additional endoscopic procedure proposed after the index EGD was recorded as a composite variable in the retrospective database. Therefore, although we were able to distinguish further endoscopic procedures from sectional imaging, we could not reliably provide a detailed breakdown of the individual endoscopic modalities.

## Conclusion

In hospitalized patients aged ≥75 years, unsedated EGD remains a clinically useful procedure. It yielded a macroscopic diagnosis consistent with the indication in 44% of cases and contributed to patient management in more than 80%, including 70% of patients without macroscopic findings. Tolerance was generally acceptable, and simple clinical features such as age, nutritional status, cognitive performance, and autonomy may help identify patients at higher risk of poor tolerance. These findings support an individualized approach to unsedated EGD in older patients.

## Data Availability

Deidentified data are available from the corresponding author upon reasonable request.

## Supplementary Material

Ergänzendes Material Supplementary Material

## References

[1] Peery A F Crockett S D Murphy C C Burden and cost of gastrointestinal, liver, and pancreatic diseases in the United States: update 2018 Gastroenterology 2019 156 01 2.54E13 2.72E13 10.1053/j.gastro.2018.08.063 PMC6689327 30315778

[2] Mönkemüller K Fry L C Malfertheiner P Schuckardt W Gastrointestinal endoscopy in the elderly: current issues Best Pract Res Clin Gastroenterol 2009 23 06 821 827 19942160 10.1016/j.bpg.2009.10.002

[3] World Health Organization World Health Statistics 2021: Monitoring Health for the SDGs, Sustainable Development Goals [Internet] Geneva World Health Organization 2021[cité 28 juin 2022]. Disponible sur:https://apps.who.int/iris/handle/10665/342703

[4] Ergenç M Uprak T K Esophagogastroduodenoscopy in patients aged 75 years and older: a single-center study Cureus 2022 14 02 e21846 35291530 10.7759/cureus.21846 PMC8896878

[5] Clarke G A Jacobson B C Hammett R J Carr-Locke D L The indications, utilization and safety of gastrointestinal endoscopy in an extremely elderly patient cohort Endoscopy 2001 33 07 580 584 11473328 10.1055/s-2001-15313

[6] Laporte M Villalon L Thibodeau J Payette H Validity and reliability of simple nutrition screening tools adapted to the elderly population in healthcare facilities J Nutr Health Aging 2001 5 04 292 294 11753498

[7] Katz S Downs T D Cash H R Grotz R C Progress in development of the index of ADL Gerontologist 1970 10 01 20 30 5420677 10.1093/geront/10.1_part_1.20

[8] Kalafat M Hugonot-Diener L Poitrenaud J Standardisation et étalonnage français du “mini-mental state” (MMS) version GRECORev Neuropsychol2003 13

[9] Derouesne C Poitreneau J Hugonot L Kalafat M Dubois B Laurent B Mini-mental state examination: a useful method for the evaluation of the cognitive status of patients by the clinician. Consensual French version Presse Med Paris 1999 28 21 1141 1148 10399508

[10] Arevalo-Rodriguez I Smailagic N Roqué-Figuls M Mini-mental state examination (MMSE) for the early detection of dementia in people with mild cognitive impairment (MCI) Cochrane Database Syst Rev 2021 7 07 CD010783 34313331 10.1002/14651858.CD010783.pub3 PMC8406467

[11] Charlson M Szatrowski T P Peterson J Gold J Validation of a combined comorbidity index J Clin Epidemiol 1994 47 11 1245 1251 7722560 10.1016/0895-4356(94)90129-5

[12] Bjerrum L Rosholm J U Hallas J Kragstrup J Methods for estimating the occurrence of polypharmacy by means of a prescription database Eur J Clin Pharmacol 1997 53 01 7 11 9349923 10.1007/s002280050329

[13] Masnoon N Shakib S Kalisch-Ellett L Caughey G E What is polypharmacy? A systematic review of definitions BMC Geriatr 2017 17 01 230 29017448 10.1186/s12877-017-0621-2 PMC5635569

[14] R: The R Project for Statistical Computing [Internet]. [cité 28 juill 2017]. Disponible sur:https://www.r-project.org/

[15] Buri L Zullo A Hassan C Upper GI endoscopy in elderly patients: predictive factors of relevant endoscopic findings Intern Emerg Med 2013 8 02 141 146 21538157 10.1007/s11739-011-0598-3

[16] Froehlich F Repond C Müllhaupt B Is the diagnostic yield of upper GI endoscopy improved by the use of explicit panel-based appropriateness criteria? Gastrointest Endosc 2000 52 03 333 341 10968846 10.1067/mge.2000.107906

[17] Perry I E Sonu I Scarpignato C Akiyama J Hongo M Vega K J Potential proton pump inhibitor-related adverse effects Ann N Y Acad Sci 2020 1481 01 43 58 32761834 10.1111/nyas.14428

[18] de la Mora G Marcon N E Endoscopy in the elderly patient Best Pract Res Clin Gastroenterol 2001 15 06 999 1012 11866489 10.1053/bega.2001.0254

