# Supplementary material for: Clinical Impact and Tolerance of Unsedated Esophagogastroduodenoscopy in Patients Aged ≥75 Years
Source: Endosc Int Open. 2026 Jul 8;14:a29015915. doi: 10.1055/a-2901-5915 (PMC13460292; doi:10.1055/a-2901-5915)
Supplement: Supplementary file 1 [file 10-1055-a-2901-5915_29021726.pdf]

Supplementary Table 1. Baseline characteristics according to MMSE availability

Characteristic	Overall population (n=200)	MMSE unavailable (n=58)	MMSE available (n=142)	p-value
Age, mean (SD), years	86.86 (5.82)	84.69 (6.26)	87.74 (5.40)	<0.001
Women, n (%)	107 (53.5)	32 (55.2)	75 (52.8)	0.762
Charlson comorbidity index, mean (SD)	6.87 (1.89)	6.53 (2.10)	7.00 (1.78)	0.113
ADL score (/6), mean (SD)	5.08 (1.29)	5.26 (1.47)	5.01 (1.21)	0.211
IADL score (/8), mean (SD)	4.01 (2.23)	4.91 (2.35)	3.64 (2.07)	<0.001
Malnutrition, n (%)				0.850
No malnutrition	31 (15.5)	9 (15.5)	22 (15.5)	
Moderate malnutrition	92 (46.0)	25 (43.1)	67 (47.2)	
Severe malnutrition	77 (38.5)	24 (41.4)	53 (37.3)	
Polypharmacy (≥5 drugs/day), n (%)	143 (71.5)	39 (67.2)	104 (73.2)	0.394
Anticoagulants, n (%)	75 (37.5)	19 (32.8)	56 (39.4)	0.376
Antiplatelet agents, n (%)	63 (31.5)	21 (36.2)	42 (29.6)	0.360
Psychoactive drugs, n (%)	88 (44.0)	16 (27.6)	72 (50.7)	0.003
Drugs that may delay gastric emptying, n (%)	72 (36.0)	14 (24.1)	58 (40.8)	0.026
Hemoglobin, mean (SD), g/dL	10.20 (1.63)	9.94 (1.43)	10.30 (1.70)	0.163
C-reactive protein, mean (SD), mg/L	42.47 (50.25)	47.86 (52.41)	40.23 (49.35)	0.337

Values are presented as mean (SD) or n (%). Comparisons were performed using Student's t-test for continuous variables and χ^2 or Fisher's exact test for categorical variables, as appropriate. ADL, Activities of Daily Living; IADL, Instrumental Activities of Daily Living; MMSE, Mini-Mental State Examination.

Supplementary Table 2. Baseline and procedural characteristics according to EGD completion

**Characteristic**	**Overall population (n=200)**	**Incomplete EGD (n=33)**	**Completed EGD (n=167)**	**p-value**
Age, mean (SD), years	86.86 (5.82)	87.58 (5.63)	86.71 (5.86)	0.428
Women, n (%)	107 (53.5)	17 (51.5)	90 (53.9)	0.802
Charlson comorbidity index, mean (SD)	6.87 (1.89)	7.24 (1.46)	6.79 (1.95)	0.132
ADL score (/6), mean (SD)	5.08 (1.29)	5.12 (1.43)	5.07 (1.26)	0.855
IADL score (/8), mean (SD)	4.01 (2.23)	3.64 (2.34)	4.08 (2.20)	0.317
MMSE available, n (%)	142 (71.0)	24 (72.7)	118 (70.7)	0.811
MMSE score, mean (SD)*	20.77 (6.01)	21.79 (6.06)	20.56 (6.00)	0.370
Malnutrition, n (%)				0.234
No malnutrition	31 (15.5)	2 (6.1)	29 (17.4)	
Moderate malnutrition	92 (46.0)	18 (54.5)	74 (44.3)	
Severe malnutrition	77 (38.5)	13 (39.4)	64 (38.3)	
Polypharmacy (≥5 drugs/day), n (%)	143 (71.5)	25 (75.8)	118 (70.7)	0.553
Anticoagulants, n (%)	75 (37.5)	7 (21.2)	68 (40.7)	0.034
Antiplatelet agents, n (%)	63 (31.5)	11 (33.3)	52 (31.1)	0.804
Psychoactive drugs, n (%)	88 (44.0)	14 (42.4)	74 (44.3)	0.842
Drugs that may delay gastric emptying, n (%)	72 (36.0)	14 (42.4)	58 (34.7)	0.400
Hemoglobin, mean (SD), g/dL	10.20 (1.63)	10.45 (1.91)	10.15 (1.57)	0.405
C-reactive protein, mean (SD), mg/L	42.47 (50.25)	43.97 (49.93)	42.18 (50.46)	0.854
Endoscopist-rated tolerance, n (%)				<0.001
Good	94 (47.0)	2 (6.1)	92 (55.1)	
Intermediate	30 (15.0)	5 (15.2)	25 (15.0)	
Poor	59 (29.5)	21 (63.6)	38 (22.8)	

Characteristic	Overall population (n=200)	Incomplete EGD (n=33)	Completed EGD (n=167)	p-value
Missing/NC	17 (8.5)	5 (15.2)	12 (7.2)	

Values are presented as mean (SD) or n (%). Comparisons were performed using Student's t-test for continuous variables and χ^2 or Fisher's exact test for categorical variables, as appropriate. *MMSE score was calculated among patients with available MMSE data. ADL, Activities of Daily Living; EGD, esophagogastroduodenoscopy; IADL, Instrumental Activities of Daily Living; MMSE, Mini-Mental State Examination; NC, not classified.

Supplementary Table 3. Multivariable logistic regression model for poor tolerance

Variable	Adjusted OR	95% CI	p-value
Age, per 5-year increase	1.30	0.89-1.88	0.171
Male sex	0.57	0.26-1.25	0.161
MMSE score, per 1-point increase	0.93	0.87-1.00	0.048
Severe malnutrition	2.49	1.13-5.52	0.024
ADL score, per 1-point increase	0.82	0.59-1.14	0.241

The model was restricted to patients with complete data for all included variables (n=142; 45 events of poor tolerance). Variables were selected a priori based on clinical relevance and univariate findings. ADL, Activities of Daily Living; CI, confidence interval; MMSE, Mini-Mental State Examination; OR, odds ratio.
